# The Predictability of Transverse Changes in Patients Treated with Clear Aligners

**DOI:** 10.3390/ma16051910

**Published:** 2023-02-25

**Authors:** Vincenzo D’Antò, Rosa Valletta, Luigi Di Mauro, Francesco Riccitiello, Robertas Kirlis, Roberto Rongo

**Affiliations:** 1 School of Orthodontics, Department of Neurosciences, Reproductive Sciences and Oral Sciences, University of Naples Federico II, Via Pansini, 5, 80131 Naples, Italy; 2 School of Pediatric Dentistry, Department of Neurosciences, Reproductive Sciences and Oral Sciences, University of Naples Federico II, Via Pansini, 5, 80131 Naples, Italy; 3 Private Practice VIC Clinic, 03162 Vilnius, Lithuania

**Keywords:** aligner treatment, expansion, predictability, accuracy

## Abstract

Arch expansion might be used to correct buccal corridors, improve smile aesthetics, resolve dental cross bite, and gain space to resolve crowding. In clear aligner treatment, the predictability of the expansion is still unclear. The purpose of this study was to evaluate the predictability of dentoalveolar expansion and molar inclination with clear aligners. In the study, 30 adult patients (27 ± 6.1 years old) treated with clear aligners were selected (treatment time: 8.8 ± 2.2 months). The upper and lower arch transverse diameters were measured for canines, first and second premolars, and first molars on two different sides (gingival margins and cusp tips); moreover, molar inclination was measured. A paired t-test and Wilcoxon test were used to compare prescription (planned movement) and achieved movement. In all cases, except for molar inclination, a statistically significant difference was found between achieved movement and prescription (*p* < 0.05). Our findings showed a total accuracy of 64% for the lower arch, 67% at the cusp level, and 59% at the gingival level, with a total accuracy of 67% for the upper arch, 71% at the cusp level, and 60% at the gingival level. The mean accuracy for molar inclination was 40%. Average expansion was greater at cusps of canines than for premolars, and it was lowest for molars. The expansion achieved with aligners is mainly due to the tipping of the crown rather than bodily movement of the tooth. The virtual plan overestimates the expansion of the teeth; thus, it is reasonable to plan an overcorrection when the arches are highly contracted.

## 1. Introduction

Clear aligners are thermoplastic removable orthodontic polymeric appliances programmed by means of CAD-CAM systems that can generate orthodontic movements [1]. Since their introduction as a treatment option in orthodontics, aligners have become increasing popular since they are more aesthetic than conventional fixed therapy, the dental pain related to orthodontic movements is reduced in the first days of the treatment [2], the risk of root resorption tends to be lower with clear aligners [3], and patients can maintain better oral hygiene [4,5]. Today, thanks to improvements in biomechanics, clinicians have the opportunity to use clear aligners to also treat class II and class III cases [6,7,8,9]. The use of auxiliaries and attachments, as well as the development of aligner materials with better mechanical and chemical characteristics [10,11,12], increases the possibility of achieving complex movements such as rotation or torque [13]. Although the use of clear aligner therapies is spreading worldwide and patient demand is increasing, the low predictability of some types of tooth movements seems to negatively affect clinicians when choosing this therapy, requiring at least two or three refinements [14]. According to the evidence, the discrepancy between predicted and clinical outcomes is around 50% [3,15,16,17]. In particular, pure tipping is the movement with the highest predictability (56%) [15], and root torque movement has the lowest accuracy [15,16]. The scientific literature supports the use of aligners as an alternative to fixed appliances in patients with mild-to-moderate malocclusions [3]. As reported by Malik, aligners can be considered an elective tool for malocclusion when characterized by crowding (from 1 to 5 mm), mild-to-moderate spacing, narrow arches that can be expanded [18], or cross bite [19] and for all malocclusions that do not need extractions, severe rotations, and bodily movements [3]. Arch expansion might be skeletal and should be performed during growth with both skeletal and functional effects [20,21,22] or dental effects and can be performed with brackets, clear aligners, or other appliances to improve the shape of the patient’s dental arch, correct buccal corridors when excessively present, improve smile aesthetics, resolve dental cross bite, and gain space to resolve crowding [23]. Clear aligners allow clinicians to plan arch expansion using two different tooth movements: buccal tipping of the dental crown (increasing the angulation of the tooth) or the translation of the tooth, which also includes root displacement. Buccal tipping of the crown is achieved when the pressure zones of the aligners, which push and determine tooth displacement, pass below the center of resistance, generating a movement [15]. On the other hand, the expansion obtained by tooth translation is among the least predictable movements because it includes root displacement [3,24]. Tooth bodily movement can be achieved with clear aligners using rectangular or ellipsoid attachments on the buccal side in order to modify aligner geometries, improving root control [15,24]. The contact of the attachment with the aligners during the dental movement generates a pseudocouple of forces, producing a counterbalancing moment. Many authors have shown that with clear aligners, the expansion is achieved more by dental tipping than bodily translation [25,26,27,28,29]. The reported accuracy of upper arch expansion ranges between 41% [30] and more than 75–80% according the most recent studies by Houle et al., Bèrnardez et al., and Burruezo et al. [25,31,32]. For the lower arch, the reported predictability is slightly higher than that for the upper arch (87.7% [25], 90% [31]). Therefore, the aim of the present study is to assess the predictability of the upper first molar inclination when expansion is performed.

## 2. Materials and Methods

The study protocol complied fully with the principles of the Declaration of Helsinki and was approved by the Ethics Committee of the University Federico II (352/21).

### 2.1. Inclusion/Exclusion Criteria

This prospective longitudinal study included 30 patients (18 females, 12 males; mean age, 27 ± 6.1 years) and 34 digital models (19 maxillary and 19 mandibular) according to the following inclusion criteria: adult patients in permanent dentition >17 y.o.; and treatment did not require extractions; no auxiliary devices during arch expansion treatment, such as crisscross elastics, bands, quadhelix/skeletal expanders, or temporary anchorage devices. Patients with syndromes, cleft palates, or pharmacological treatment that may affect tooth movement (bisphosphonates or prostaglandin inhibitors) were excluded from the study. To ensure clinical irrelevance, patients with Prescriptions for linear movements less than 0.5 mm and angular movements less than 2° were excluded from the statistical analysis.

### 2.2. Treatment Protocol

All patients were treated exclusively with Ordoline aligners (UAB Ordoline, Vilnius, Lithuania).

Patients wore aligners full-time for a minimum of 22 h a day, except during meals and during oral hygiene procedures.

The orthodontic treatment setup and the staging of the aligners were planned according to the following tooth movement limits for each aligner: linear displacements (arch expansion) of 0.25 mm and 2° for buccal-lingual inclination.

Aligners were changed every 10 days.

### 2.3. Data Collection 

For each upper and lower arch, three digital models (stereolithography/STL files) were analyzed—digital model at the beginning of treatment (T0), digital model at the end of the first set of aligners (T2; treatment time, 8.8 ± 2.2 months), and the virtual planned digital model (T1)—reflecting the treatment outcome simulated with the planning software. The digital scans (T0 and T2) were acquired by means of an intraoral scanner (IOS).

### 2.4. Superimposition Method 

**Figure 1:**
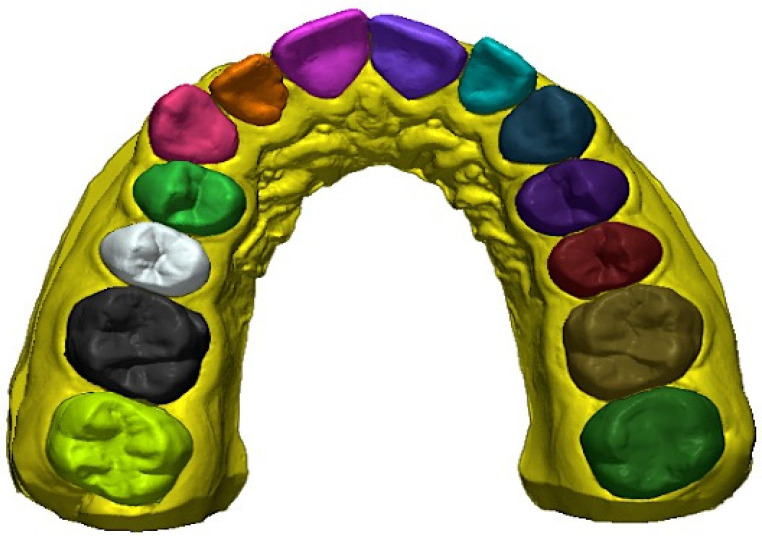
Tooth segmentation.

The STL files (T0, T1, and T2), were imported into Geomagic Control X (3D Systems, Rock Hill, SC, USA), a 3D metrology software. Digital models were imported in pairs: first, T0 − T1 were compared to measure the amount of planned movement; then, T0 − T2 were compared to establish the amount of obtained movement. T0 was always designated as “reference data”, and T1 or T2 were designated as “measured data”. The pretreatment digital model (T0) was partially segmented by isolating individual teeth, from canine to first molar on both sides (left and right) (Figure 1). The segmentation allowed us to perform a partial surface-based best fit with 50 iterations for each tooth in order to identify the same landmarks in the .stl files that would be used to obtain the linear measurements (transverse diameters). To obtain the angular measurement (molar inclination), the occlusal reference plane [33] was defined on T0. Then, for each pair of digital models (first, T0 − T1, then T0 − T2), an initial superimposition by means of a “3-point alignment” (based on the mesiobuccal cusp tips of the first molars and on the mesial-incisal point of the right central incisor) was performed, followed by a global best-fit registration with 50 iterations. 

**Figure 2:**
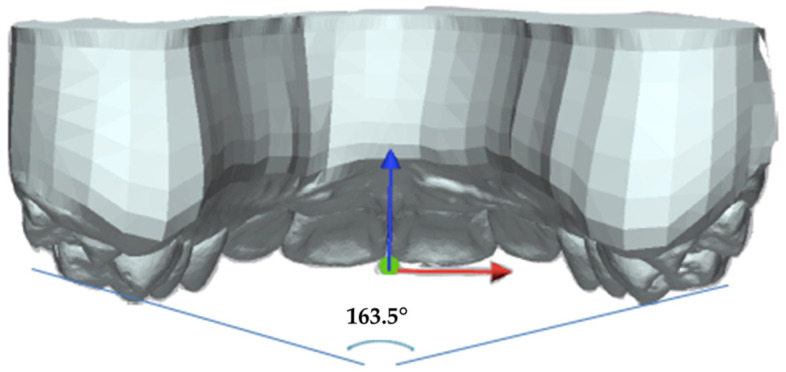
Molar inclination.

Once the models were superimposed, the plane perpendicular to the occlusal reference was defined to project the vectors that define the molar inclination angle (Figure 2).

A coordinate reference system was created with the XY plane as the transversal plane, the XZ plane as the sagittal plane, and the YZ plane as the coronal plane.

### 2.5. Transverse Parameters and Molar Inclination

The superimposition protocol allowed the landmarks to be placed in the same position for each digital model, obtaining comparable measurements. A total of 14 landmarks (10 on the buccal side and 8 on the gingival side) were chosen for each digital model [28,34] as follows:

- Buccal cusp tips of canines and premolars;
- Mesiovestibular and distovestibular cusp tips of the first molars;
- The center of the gingival surface of canines and premolars in contact with the mucosa;
- The gingival point corresponding to the groove of the first molars in contact with the mucosa.

**Figure 3:**
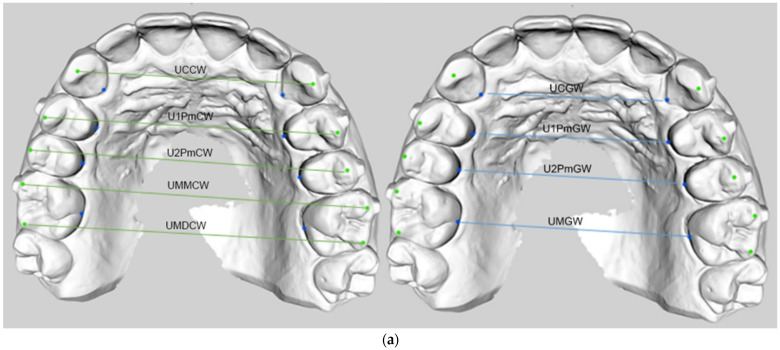

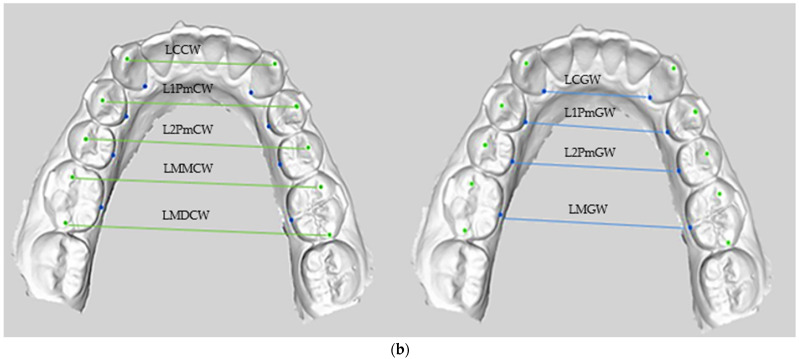
Landmarks and transverse parameters on the buccal and gingival side. (**a**) Upper arch transverse diameters. Upper canine gingival width (UCGW); upper first premolar gingival width (U1PmGW); upper second premolar gingival width (U2PmGW); upper first molar gingival width (UMGW); upper canine cusp width (UCCW); upper first premolar cusp width (U1PmCW); upper second premolar cusp width (U2PmCW); upper first molar mesiobuccal cusp width (UMMCW); upper first molar distobuccal cusp width (UMDCW). (**b**) Lower arch transverse diameters. Lower canine gingival width (LCGW); lower first premolar gingival width (L1PmGW); lower second premolar gingival width (L2PmGW); lower first molar gingival width (LMGW); lower canine cusp width (LCCW); lower first premolar cusp width (L1PmCW); lower second premolar cusp width (L2PmCW); lower first molar mesiobuccal cusp width (LMMCW); lower first molar distobuccal cusp width (LMDCW).

The landmarks were connected via digital caliper in Geomagic Control X software to obtain the following diameters (Figure 3):

- Upper canine gingival width (UCGW): distance between the center of the gingival surfaces of canines in contact with the palatal mucosa;
- Upper first premolar gingival width (U1PmGW): distance between the center of the gingival surfaces of the first premolars in contact with the palatal mucosa;
- Upper second premolar gingival width (U2PmGW): distance between the center of the gingival surfaces of the second premolars in contact with the palatal mucosa;
- Upper first molar gingival width (UMGW): distance between the grooves of the first molars in contact with the palatal mucosa;
- Upper canine cusp width (UCCW): distance between the buccal cusp tips of canines;
- Upper first premolar cusp width (U1PmCW): distance between the buccal cusp tips of the first premolars;
- Upper second premolar cusp width (U2PmCW): distance between the buccal cusp tips of the second premolars;
- Upper first molar mesiobuccal cusp width (UMMCW): distance between the mesiobuccal cusp tips of the first molars;
- Upper first molar distobuccal cusp width (UMDCW): distance between the distobuccal cusp tips of the first molars;
- Lower canine gingival width (LCGW): distance between the center of the gingival surfaces of canines in contact with the lingual mucosa;
- Lower first premolar gingival width (L1PmGW): distance between the center of the gingival surfaces of the first premolars in contact with the lingual mucosa;
- Lower second premolar gingival width (L2PmGW): distance between the center of the gingival surfaces of the second premolars in contact with the lingual mucosa;
- Lower first molar gingival width (LMGW): distance between the grooves of the first molars in contact with the lingual mucosa;
- Lower canine cusp width (LCCW): distance between the buccal cusp tips of canines;
- Lower first premolar cusp width (L1PmCW): distance between the buccal cusp tips of the first premolars;
- Lower second premolar cusp width (L2PmCW): distance between the buccal cusp tips of the second premolars;
- Lower first molar mesiobuccal cusp width (LMMCW): distance between the mesiobuccal cusp tips of the first molars;
- Lower first molar distal cuspid width (LMDCW): distance between the distobuccal cusp tips of the first molars.

Molar inclination was measured as the angle formed by the intersection of the vectors passing through the distobuccal and mesiolingual cusps of both maxillary first molars and projected onto the coronal plane (Figure 2). 

Hence, for all the diameters, intercanine distance, interpremolar distances, and intermolar distance for upper and lower arch, measurements were taken at both gingival and cusp height.

### 2.6. Prescription, Achieved Movement, and Accuracy

**Figure 4:**
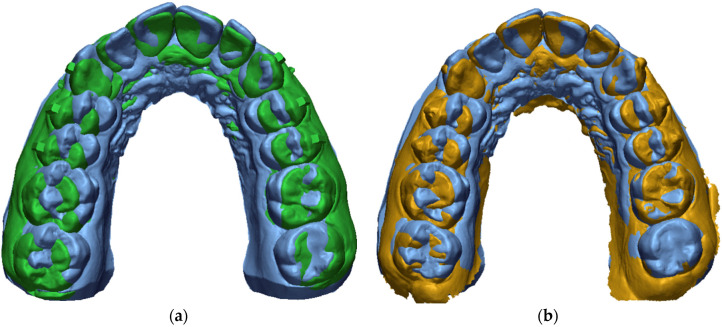
Superimposition example. (**a**) Superimposition of T0 (blue scan) and T1 (green scan). (**b**) Superimposition of T0 (blue scan) and T2 (yellow scan).

Molar inclination and transverse diameters (on both gingival and cusp sides) were analyzed by assessing the following:

- Prescription (planned movement): T1 − T0 (difference between T1 measurements and T0 measurements) (Figure 4);
- Achieved movement: T2 − T0 (difference between T2 measurements and T0 measurements) (Figure 4);
- Accuracy (predictability): (T2 − T0)/(T1 − T0) % (the amount of movement that clinically occurred compared with the movement planned in virtual plan, expressed in percentage). Furthermore, total accuracy was calculated as the mean of the accuracy of all the teeth for each arch.

### 2.7. Statistical Analysis

Considering the first molar MV cusp width as the main outcome, an effect size of 1 was calculated in a previous study [25]. A sample size of 10 digital models was needed using a paired Student’s *t*-test with an alpha error of 0.05 to achieve 80% power. Descriptive statistical analysis included means, standard deviations, and 95% CI for prescription, achieved movement, and accuracy. The significance level was set at 0.05. A Shapiro–Wilk normality test was used to assess the normal distribution of the data. In the case of a normal distribution, differences between obtained movements (T2 − T0) and prescribed movements (T1 − T0) were assessed by a paired Student’s *t*-test; in cases in which data were not normally distributed, the Wilcoxon test was used. *p*-values < 0.05 were considered significant. Intraexaminer and interexaminer reproducibility of the measurements were evaluated by means of the intraclass correlation coefficient. Twenty percent of the digital dental models were reanalyzed by the same operator and again by a different operator 4 weeks after the first examination. Linear movements of less than 0.5 mm and angular movements of less than 2° were excluded from the statistical analysis because they were not clinically relevant. The statistical package SPSS 22(IBM, Chicago, IL, USA) was used.

## 3. Results

### 3.1. Analysis of Intraclass Correlation Coefficient (ICC)

The reproducibility of measurements was shown by the ICC score, which was determined by the same operator at different moments and by different operators (intraexaminer and interexaminer ICC, respectively). For the intraexaminer and extraexaminer ICC, our study showed scores of 0.996 and 0.982, respectively.

### 3.2. Analysis of Prescription and Achieved Movement

Table 1 and Table 2 show means, standard deviations, upper and lower limits of 95% confidence intervals for the prescription and achieved movement, and the differences between the amount of planned and achieved expansion (T1 − T2) for each subgroup for each dental arch. 

The highest mean prescription both for lower and upper arch expansion was found at the cusps of the second premolars (2.44 mm and 3.22 mm for the lower arch and upper arch, respectively); the lowest mean prescription was found at the gingival level of the first molars (1.65 mm) for the lower arch and at the distobuccal cusps of the first molars (2.05 mm) for the upper arch.

The greatest amount of expansion was achieved at the cusps of the upper and lower first premolars (2.42 mm and 1.72 mm, respectively), while the lowest recorded value was found on the gingival side of the lower (0.82 mm) and upper canines (0.95 mm).

A minor difference between prescription and achieved movement of expansion for both the lower arch and the upper arch was found at the canine cusps (LA, 0.35 mm with a total expansion of 1.67 mm; UA 0.27 mm with a total expansion of 1.78 mm).

In every subgroup, a statistically significant difference (*p* value < 0.05) was found between the prescription and achieved movement.

### 3.3. Analysis of Accuracy

Table 3 shows means, standard deviations, and 95% CIs for upper and lower arch accuracy. Our findings show a total accuracy of 64% for the lower arch, 67% at the cusp level, and 59% at the gingival level, with a total accuracy of 67% for the upper arch, 71% at the cusp level, and 60% at the gingival level. In particular, in the lower arch, the most reliable area to predict the expansion was found to be the cusps of the canines (79%), while the lowest accuracy was found at the gingival level of the first molars (57%). Similarly, in the upper arch, the greatest accuracy was found at the cusps of canines (83%), while the predictability of expansion was lowest at the gingival level of the first molars (55%).

### 3.4. Analysis of Molar Inclination

Table 2 shows the means, standard deviations, and 95% CIs for the prescription and achieved movement, as well as the difference between the prescribed and achieved molar inclination. Table 3 reports the mean accuracy of molar inclination.

An accuracy of 40% and a non-statistically significant difference (*p* value > 0.05) was found between the prescription and obtained movement.

Our study revealed an intermolar angle reduction of 2°.

## 4. Discussion

The purpose of the present study was to evaluate the predictability of expansion with clear aligners and the changes in the inclination of the upper first molars. These data were calculated by comparing the planned orthodontic movement through the virtual setup (T1) with the patient′s digital models (T0 − T2). In the present study, landmarks were placed on both the coronal and gingival sides to differentiate between expansion achieved by coronal tipping and that achieved by tooth body movement. An adult population was chosen for this study in order to avoid bias due to the normal transverse growth effect of the upper and lower jaws. Geomagic software was used to measure linear parameters, the precision of which was demonstrated by Sousa et al. in 2012 [35] and by Adel SM et al. in 2022 [36]. In 2007, Kravitz et al. [30] studied the predictability of expansion along the anteroposterior direction, revealing a mean accuracy of 41%. In 2017, Houle et al. [25] obtained a predictability ranging from 53% at the gingival level of the molars to 88% at the vestibular level of the canines. In addition, Houle’s study reported a decrease in predictability in the posterior sectors. In 2020, Solano-Mendoza et al. [28] evaluated the accuracy of expansion of the upper arch on the gingival and buccal sides, reporting an average obtained movement of 1.38 mm and 0.54 mm at the cusp and gingival levels of the canines, respectively; 1.39 mm at the gingival level of the first premolars; 1.25 mm at the gingival level of the second premolars; and 0.56 mm at the gingival level of the molars. In 2020 and 2021, other authors [31,32] evaluated the predictability of transverse changes in arch expansion, reporting values ranging from 65% (second molars) to 81% (at the level of the second premolars), and for all linear measurements evaluated between T2 and T3 (post treatment and virtual setup), a statistically significant difference was found. The following amount average movement was obtained: 1.87 mm for canines, 3.14 mm for the first premolars, 3.45 mm for the second premolars, 2.57 mm for the first molars, and 0.45 mm for the second molars [32]. In 2020, Haouili et al. [16] published a prospective study on the predictability of orthodontic movement with aligners, and an average accuracy of 50% was reported for tooth movements with clear aligners; the highest overall accuracy occurred with a buccal crown tip (56%). Our findings revealed that the predictability of expansion ranges to range from 57% to 79% for the lower arch and from 54% to 83% for the upper arch. The results of our study confirm the scientific evidence, i.e., that the predictability of expansion is greater when assessed at the cusps of the teeth rather than at the gingival level, and there is a progressive reduction in predictability from anterior to posterior segments. The expansion at the cusps is more predictable because the aligners move the teeth by coronal tipping. Therefore, an increase in transverse diameters is possible predominantly by the tipping of the crown despite body movement, which also requires root displacement. Although clinicians can use aids such as attachments to increase system stiffness and a counterbalancing moment to control the root displacement, it is still considered unpredictable [3,24]. Instead, the reduction in the predictability from the anterior to posterior segments described by Lione et al. as a “drawbridge expansion model” [37] was mainly due to the different shapes of the teeth, the loss of tracking of the aligners when a large amount of orthodontic movement is prescribed, the loss of fitting of the aligners in the posterior sectors during the displacement of the anterior teeth, and the greater multiradicular tooth resistance to orthodontic movement. Therefore, as reported in the scientific literature, the canines and first premolars are the teeth with the highest accuracy and greatest achieved expansion, while the molars have the lowest accuracy and least achieved expansion [16,25]. The movement of canines and premolars is easier than that of other teeth because they are placed on a straight line, free of any binding occlusal contact, and the canines usually show a lower accuracy than premolars, since they are located on the arch of a circle. Thus, their movement is influenced more by the dimensions of anterior teeth [37]. Instead, the expansion of the intermolar diameter showed the lowest predictability both at gingival and cusp levels; the posterior location of the molars, the loss of fitting with anterior teeth displacements, their tendency to be curved towards the midline, and the greatest anchorage value due to their anatomy negatively influence this movement [27,32,37]. Nevertheless, in our study, the accuracy of expansion of the canines was slightly higher than that of the premolars, but it could be explained by the lower amount of expansion required in the intercanine area compared to the interpremolar area. Although our study reveals a greater predictability for the upper arch than the lower arch, the reported results highlight that in relation to a similar amount of prescribed movement, the anatomical limits of the mandibular bone, as well as the greater mineralization and occlusal stability between the upper and lower arches, could play a fundamental role in achieving the planned expansion [38]. For all the reasons previously described, the buccal tipping of the first upper molars increases when an expansion is performed. The accuracy of the molar inclination movement was 40%. However, the result of inclination of the first molars when an expansion is prescribed should be analyzed; when the required expansion was predominantly characterized by vestibular tipping, agreement between the virtual plan digital model and post treatment was found (accuracy of 83.6%). On the other hand, when the prescribed expansion implied a root torque control (vestibular radicular torque) with a consequent opening of the intermolar angle, the accuracy of the movement drastically dropped in some cases in which buccal-radicular torque was prescribed, but there was buccal coronal tipping. As reported by Zhou et al. [27], the ratio of the expansion movement between the root and the crown was approximately 2:5. For this reason, during digital planning, it is necessary to overcorrect the buccal root torque in relation to the amount and type of planned expansion [39]. 

Finally, one study compared the expansion between conventional and aligner techniques, supporting the idea of a greater expansion with self-ligating brackets [40]. However, the amount of expansion with aligners is directly correlated with the amount of planned expansion in the virtual checkup. Furthermore, different tooth movement can be supposed with multi-brackets therapy; in the first phase of the treatment with an undersized wire, there was less control of tooth movement, and dental tipping occurred, whereas control of the root position was achieved only with a full-thickness wire. On the other hand, with aligners, through virtual staging it is possible to plan from the beginning, a force balancing the vestibular crown movement to reduce the pure tipping.

Regarding the predictability of linear measurements, as for buccal root torque, a statistically significant difference was found between the planned and actual movement. Therefore, it is reasonable to also plan an overcorrection during expansion movement. In particular, it could be quite useful when there are very contracted arches or when teeth are placed in more palatal orientation and must achieve a considerable amount of expansion movement in the buccal direction.

This study is subject to some limitations. The sample size was small, but it satisfied the criteria of the sample size calculation. It was not possible to use CBCTs to assess the true root displacement and the changes in inclination of the first upper molars [39], nor was it possible to monitor patient compliance. However, the method used for superimposition is highly supported by the literature. In this paper, we introduced data on a new brand of aligners, and all the superimpositions were performed at the end of the first set of aligners. Only one brand of aligners was included in the study, so it was not possible to compare the achieved results with those of other brands of aligners, and results are related only to the Ordoline aligners.

## 5. Conclusions

In this study conducted with Ordoline aligners, the mean accuracy of dentoalveolar upper arch expansion was 71% at the cusps and 60% at the gingival level, which is similar to the mean accuracy of dentoalveolar lower arch expansion, which was 67% at the cusps and 59% at the gingival level. 

The amount of the movement achieved was higher for canines than for premolars, and it was lowest for molars. When expansion is required, aligners increase molar inclination. Thus, the expansion achieved with aligners is mainly due to the tipping of the crown rather than bodily movement of the tooth, which also includes root displacement.

Aligners do not achieve 100% of the prescription; thus, constant monitoring and an overcorrection of the expansion might be recommended.

## Figures and Tables

**Table 1:** Means, standard deviations, upper and lower limits of 95% confidence intervals for the prescription and achieved movement, and differences between the amount of expansion prescribed and achieve for the lower arch. All measurements are in millimeters (mm). * Indicates that Wilcoxon test was used.

		Prescription		Achieved Movement		|AM-P|		AM vs. *p*
	n	Mean ± SD	95% CI LL-UL	Mean ± SD	95% CI LL-UL	Mean ± SD	95% CI LL-UL	*p*-Value
CCW	17	1.67 ± 0.93	1.20–2.15	1.28 ± 0.53	1.01–1.56	0.35 ± 0.44	0.12–0.58	0.0025 *
CGW	17	1.57 ± 0.69	1.21–1.92	0.82 ± 0.24	0.70–0.95	0.63 ± 0.62	0.31–0.94	<0.001
1°PmCW	19	2.34 ± 0.88	1.89–2.79	1.72 ± 0.63	1.39–2.04	0.56 ± 0.46	0.32–0.80	<0.001
1°PmGW	17	1.85 ± 0.73	1.48–2.23	1.03 ± 0.40	0.82–1.23	0.61 ± 0.61	0.30–0.92	<0.001
2°PmCW	18	2.44 ± 0.65	2.10–2.77	1.43 ± 0.34	1.26–1.61	0.90 ± 0.77	0.50–1.29	<0.001
2°PmGW	17	2.08± 0.67	1.73–2.43	1.24 ± 0.51	0.98–1.50	0.75 ± 0.59	0.45–1.06	<0.001 *
1°MMVW	18	1.95 ± 0.65	1.61–2.29	1.20 ± 0.46	0.96–1.44	0.59 ± 0.52	0.32–0.86	<0.001 *
1°MDVW	17	1.69 ± 0.72	1.32–2.06	0.94 ± 0.39	0.74–1.14	0.67 ± 0.52	0.40–0.94	<0.001
1°MGW	16	1.65 ± 0.88	1.20–2.11	0.83 ± 0.31	0.67–0.99	0.69 ± 0.76	0.30–1.08	<0.001

**Table 2:** Means, standard deviations, upper and lower limits of 95% confidence intervals for the prescription and achieved movement, and differences between the amount of expansion and molar inclination prescribed and achieved for the upper arch. All measurements are in millimeters (mm). * Indicates that Wilcoxon test was used.

		Prescription		Achieved Movement		|AM-P|		AM vs. *p*
	n	Mean ± SD	95% CI LL-UL	Mean ± SD	95% CI LL-UL	Mean ± SD	95% CI LL-UL	*p*-Value
CCW	16	1.78 ± 0.78	1.37–2.18	1.46 ± 0.64	1.13–1.78	0.27 ± 0.28	0.13–0.41	<0.001
CGW	16	1.59 ± 0.43	1.37–1.81	0.95 ± 0.18	0.85–1.04	0.54 ± 0.41	0.33–0.75	<0.001
1°PmCW	18	3.14 ± 1.50	2.37–3.91	2.42 ± 1.19	1.81–3.04	0.71 ± 0.44	0.49–0.94	<0.001 *
1°PmGW	18	2.19 ± 0.86	1.75–2.63	1.21 ± 0.38	1.01–1.40	0.99 ± 0.60	0.68–1.29	<0.001
2°PmCW	18	3.22 ± 1.87	2.26–4.18	2.17 ± 1.04	1.63–2.70	1.05 ± 0.94	0.57–1.53	<0.001
2°PmGW	18	2.29 ± 1.05	1.75–2.82	1.36 ± 0.58	1.06–1.66	0.88 ± 0.74	0.49–1.26	<0.001
1°MMVW	17	2.57 ± 1.24	1.93–3.21	1.43 ± 0.51	1.17–1.70	1.08 ± 0.96	0.58–1.57	<0.001
1°MDVW	17	2.05 ± 0.82	1.63–2.47	1.17 ± 0.45	0.94–1.40	0.83 ± 0.53	0.56–1.11	<0.001 *
1°MGW	16	2.13 ± 0.87	1.68–2.58	1.00 ± 0.31	0.84–1.16	1.07 ± 0.74	0.69–1.45	<0.001
M.I.	15	−0.82 ± 6.65	−4.24–−1.37	−2.00 ± 2.82	−2.96–−0.06	0.57 ± 4.21	−1.59–−2.74	>0.05

**Table 3:** Means, standard deviations, and upper and lower limits of 95% confidence intervals for accuracy in lower and upper arch.

	Lower Arch		Upper Arch	
	Mean ± SD	95% CI LL-UL	Mean ± SD	95% CI LL-UL
CCW	79.90 ± 11.1%	74.25–86.55%	82.79 ± 12.25%	76.49–89.09%
CGW	59.78 ± 26.13%	46.34–73.22%	61.92 ± 12.91%	55.28–68.56%
1°PmCW	75.22 ± 12.42%	68.83–81.60%	78.36 ± 10%	73.23–83.50%
1°PmGW	60.39 ± 21.30%	49.43–71.34%	57.13 ± 11.89%	51.02–63.25%
2°PmCW	61.95 ± 19.65%	51.84–72.05%	73.84 ± 15.64%	65.80–81.89%
2°PmGW	61.16 ± 19.65%	51.05–71.27%	62.57 ± 16.73%	53.97–71.18%
1°MMVW	63.39 ± 17.69%	54.29–72.49%	60.78 ± 16.29%	52.40–69.16%
1°MDVW	58.01 ± 14.72%	50.44–65.58%	60.40 ± 14.82%	52.78–68.03%
1°MGW	57.50 ± 21.05%	46.68–68.32%	53.18 ± 21.42%	42.16–64.20%
M.I.			40.03 ± 53.47%	11.20–65.97%

## Data Availability

The data presented in this study are available upon request from the corresponding author.
